# Nonalcoholic fatty liver disease test: an external validation cohort

**DOI:** 10.1007/s42000-023-00502-1

**Published:** 2023-11-13

**Authors:** Stergios A. Polyzos, Apostolis Papaefthymiou, Michael Doulberis, Jannis Kountouras

**Affiliations:** 1 First Laboratory of Pharmacology, School of Medicine, Aristotle University of Thessaloniki, 54124 Thessaloniki, Macedonia Greece; 2 Pancreaticobiliary Medicine Unit, University College London Hospitals (UCLH), London, UK; 3 Gastroklinik, Private Gastroenterological Practice, Horgen, Switzerland; 4 Division of Gastroenterology and Hepatology, Medical University Department, Kantonsspital Aarau, Aarau, Switzerland; 5 Second Medical Clinic, School of Medicine, Aristotle University of Thessaloniki, Ippokration Hospital, Thessaloniki, Macedonia Greece

**Keywords:** CHA index, Index of NASH, NAFLD test, Nonalcoholic fatty liver disease, Nonalcoholic steatohepatitis, Non-invasive index

## Abstract

**Purpose:**

Non-invasive diagnosis of nonalcoholic fatty liver disease (NAFLD) and its advanced phenotypes (e.g., nonalcoholic steatohepatitis; NASH) is a hot research topic. The aim of this report was the validation of a novel non-invasive index of NAFLD, the “NAFLD test,” recently introduced for the diagnosis of NAFLD (vs. non-NAFLD controls).

**Methods:**

This was a post-hoc analysis of a previous study. The NAFLD test was calculated in NAFLD patients and non-NAFLD controls; the performance of the test was compared with that of other non-invasive indices of NAFLD (fatty liver index [FLI] and hepatic steatosis index [HSI]), and other indices of NASH (index of NASH [ION] and cytokeratin-18/homeostasis model assessment-insulin resistance/aspartate transaminase index [CHAI]).

**Results:**

The NAFLD test was higher in NAFLD patients than in controls (1.89 ± 0.14 vs. 1.30 ± 0.06, respectively; *p* < 0.001). In NAFLD patients, the NAFLD test was higher in NASH patients than in those with simple nonalcoholic fatty liver (NAFL) (2.21 ± 0.24 vs. 1.57 ± 0.08, respectively; *p* = 0.007). The area under the receiver operating characteristic curve (AUC) of the NAFLD test was 0.84 (95% CI: 0.74–0.94; *p* < 0.001) for differentiation between NAFLD and non-NAFLD controls and its performance was similar to that for FLI and HSI. For differentiation between NASH and NAFL patients, the AUC of the NAFLD test was 0.88 (95% CI: 0.62–0.96; *p* = 0.007) and its performance was superior to that for ION and CHAI.

**Conclusions:**

The NAFLD test was validated in this external cohort for the non-invasive diagnosis of NAFLD patients vs. non-NAFLD individuals. It was also shown to differentiate between NASH and NAFL patients with acceptable accuracy.

## Introduction

Nonalcoholic fatty liver disease (NAFLD) remains a highly prevalent disease (25–30% of the general population) without to date any approved pharmaceutical treatment [1]. The prevalence of the disease is higher in specific groups, such as in patients with type 2 diabetes mellitus (T2DM), being 68% in Europe [2]. Owing to its close association with T2DM, obesity, and metabolic syndrome, a multi-society Delphi consensus statement has recently recommended changing its nomenclature to metabolic dysfunction-associated steatotic liver disease (MASLD), a change accompanied by different criteria, resembling those of the metabolic syndrome [3].

Liver biopsy is the gold standard for the staging and grading of the disease, although it is difficult to perform in all NAFLD patients given both the high prevalence of the disease and the limitations of the method (i.e., invasiveness, low acceptance by patients, potential complications, and sampling variability) [4]. Consequently, non-invasive diagnostic tests for NAFLD and more advanced phenotypes of the disease, i.e., nonalcoholic steatohepatitis (NASH) and hepatic fibrosis, are a hot topic of relevant research [5, 6]. In this regard, many groups have introduced non-invasive indices for the diagnosis of NAFLD (vs. individuals without NAFLD), or of NASH (vs. NAFL), or of significant or advanced fibrosis (vs. no or early fibrosis), as elsewhere reviewed in detail [7]. Ideally, accurate indices are needed for both the diagnosis and long-term follow-up of patients with NAFLD, similarly to glycated hemoglobin used for the diagnosis and follow-up of patients with diabetes [8, 9]. Recently, a novel non-invasive index of NAFLD, the “NAFLD test” was introduced, which showed acceptable accuracy in three different populations (Egyptian, Chinese, and Chilean) for the diagnosis of NAFLD (vs. non-NAFLD) [10].

In this report, we aimed to validate the diagnostic accuracy of the NAFLD test in an external population.

## Patients and methods

This was a post-hoc analysis of a previous, single-center, case–control study [11]. Inclusion criteria for the NAFLD patients were the following: (a) age > 18 years; (b) liver ultrasonography indicating fatty liver and abnormal liver function tests for at least 6 months before liver biopsy; and (c) performance of liver biopsy. Participants of similar age, sex, and body mass index (BMI), who were apparently healthy individuals undergoing a regular check-up for professional needs, were recruited as controls. Inclusion criteria for the controls were the following: (a) age > 18 years; (b) no history of abnormal liver ultrasound imaging or abnormal liver function tests; and (c) currently normal liver ultrasonography and normal liver function tests. Liver biopsy was not performed in the controls for obvious ethical considerations. Exclusion criteria were the same for patients and controls with the aim of excluding secondary causes of fatty liver, including alcohol consumption, hepatitis B and C, autoimmune hepatitis, drug-induced hepatitis, etc., as previously described in detail [11]. NASH-related cirrhosis (F4) was also an exclusion criterion. The study protocol was in accordance with the 1975 Declaration of Helsinki and was approved by the ethics committee of the School of Medicine, Aristotle University of Thessaloniki, Greece.

Physical examination and blood sampling were performed at 8:00–9:00 am after overnight fasting and 1–2 h prior to liver biopsy, which was performed under computerized tomography guidance. The staging and grading of NAFLD was based on the criteria of the NASH Clinical Research Network classification. Serum alanine transaminase (ALT), total cholesterol, and other biochemical tests were measured on an automated biochemical analyzer (Olympus AU2700; Olympus, Hamburg, Germany) [11]; high-sensitivity C-reactive protein (CRP) was measured with the latex-enhanced immunonephelometric assay on a BNII analyzer (Siemens Healthcare Diagnostics, Deerfield, IL, USA; total coefficient of variation 4.0–5.0%).

BMI was calculated with the formula: body weight (kg)/height^2^ (m^2^). The NAFLD test was calculated with the formula -0.695 + 0.031 × BMI (kg/m^2^) + 0.003 × cholesterol (mg/dl) + 0.014 × ALT (U/l) + 0.025 × CRP (mg/l) [10]. The performance of the NAFLD test was compared with the performance of the following: (a) other previously introduced non-invasive indices of NAFLD (for the differentiation between NAFLD and non-NAFLD controls), i.e., fatty liver index (FLI) [12] and hepatic steatosis index (HSI) [13], and (b) other indices of NASH (for the differentiation between NASH and simple nonalcoholic fatty liver [NAFL]), i.e., index of NASH (ION) [14] and cytokeratin-18/homeostasis model assessment-insulin resistance (HOMA-IR)/aspartate transaminase (AST) index (CHAI) [11], following standard equations (Table 1).

**Table 1:** Equations of the comparative non-invasive indices of NAFLD

Non-invasive index	Equation	Suggested cut-offs
*Indices of NAFLD (differentiation between NAFLD and non-NAFLD controls)*		
FLI [12]	[(e^0.953×ln(TG, mg/dl) +0.139×BMI (kg/m2) +0.718×ln(GGT, U/l) +0.053×WC (cm) −15.745^) / (1 + e^0.953×ln(TG, mg/dl) +0.139×BMI (kg/m2) +0.718×ln(GGT, U/l) +0.053×WC (cm) −15.745^)] × 100	< 30: rule out NAFLD ≥ 60: rule in NAFLD
HSI [13]	8 × ALT (U/l)/AST (U/l) + BMI (kg/m^2^) + 2 (if female) + 2 (if presence of T2DM)	< 30: rule out NAFLD ≥ 36: rule in NAFLD
*Indices of NASH (differentiation between NASH and NAFL)*		
CHAI [11]	AST (U/l) × HOMA-IR × CK-18 (U/l) / 1000	na
ION [14]	*Males:* 1.33 × WC (cm)/HC (cm) + 0.03 × TG (mg/dl) + 0.18 × ALT (U/l) + 8.53 × HOMA-IR—13.93 *Females:* 0.02 × TG (mg/dl) + 0.24 × ALT (U/l) + 9.61 × HOMA-IR—13.99	< 50: rule out NASH ≥ 50: rule in NASH

Abbreviations: *ALT* alanine transaminase; *AST* aspartate transaminase; *CHAI* cytokeratin-18/HOMA-IR/AST index; *CK-18* cytokeratin-18; *BMI* body mass index; *FLI* fatty liver index; *GGT* gamma-glutamyl transferase; *HC* hip circumference; *HSI* hepatic steatosis index; *HOMA-IR* homeostasis model assessment—insulin resistance; *ION* index of nonalcoholic steatohepatitis; *NAFL* nonalcoholic fatty liver (simple steatosis); na, not available; *NAFLD* nonalcoholic fatty liver disease; *NASH* nonalcoholic steatohepatitis; *T2DM* type 2 diabetes mellitus; *TG* triglycerides; *WC* waist circumference

### Statistical analysis

Continuous variables were presented as mean ± standard error of the mean (SEM), if normally distributed, or as median (interquartile range), if not normally distributed. Categorical variables were presented as absolute and/or relative frequencies. The Kolmogorov–Smirnov test was used to check the normality of distributions of continuous variables. The independent samples t-test or Mann–Whitney test were used for between group comparisons of continuous variables. The chi-square or Fischer exact test was used for between group comparisons of categorical variables. Receiver operating characteristic (ROC) curve analysis was used and the area under the ROC curve (AUC) was calculated to test the performance of the NAFLD test and other previously introduced non-invasive tests. Statistical analysis was performed with SPSS 29.0 for Macintosh (IBM Corp., Armonk, NY).

## Results

**Fig. 1:**
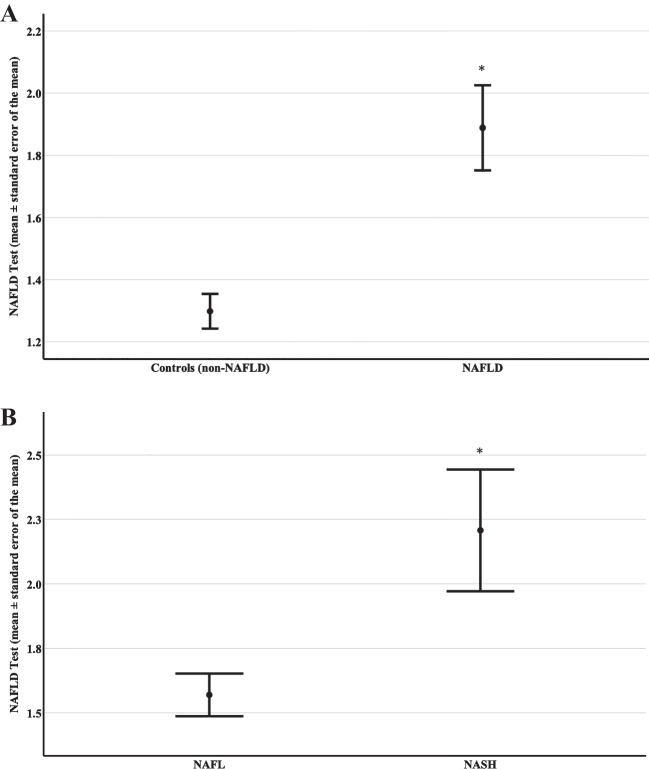
**A** NAFLD test (mean ± standard error of the mean) in individuals without NAFLD (controls; non-NAFLD) and patients with NAFLD. The NAFLD test was higher in NAFLD patients than in non-NAFLD individuals. The Mann–Whitney test was used for the comparison between groups. *: *p* < 0.001 compared to control group. **B** NAFLD test (mean ± standard error of the mean) in patients with NAFL (simple steatosis) and patients with NASH. The NAFLD test was higher in NASH than in NAFL patients. The Mann–Whitney test was used for the comparison between groups. *: *p* = 0.007 compared to NAFL group. Abbreviations: NAFL, nonalcoholic fatty liver; NAFLD, nonalcoholic fatty liver disease; NASH, nonalcoholic steatohepatitis

Thirty patients (22 women) with biopsy-proven NAFLD (15 with NAFL and 15 with NASH) and 24 controls (20 women) were included in this study. Comparative data of the study groups were presented in detail in the initially published article [11]. Regarding the variables included in the formula of the NAFLD test, the data between patients and controls, respectively, were the following: BMI (kg/m^2^) 30.6 (29.6—36.7) vs. 30.0 (28.8 – 31.8) (*p* = 0.077); cholesterol (mg/dl) 214 ± 7 vs. 226 ± 8 (*p* = 0.282); ALT (U/l) 45.5 (30.3 – 60.0) vs. 17.0 (13.0 – 23.0) (*p* < 0.001); and CRP (mg/l) 4.8 ± 0.9 vs. 3.9 ± 0.4 (*p* = 0.309). The NAFLD test was higher in NAFLD patients than in controls (1.89 ± 0.14 vs. 1.30 ± 0.06; *p* < 0.001; Fig. 1A). In NAFLD patients, the NAFLD test was higher in the NASH than in the NAFL subgroup (2.21 ± 0.24 vs. 1.57 ± 0.08; *p* = 0.007; Fig. 1B). The NAFLD test was not different between NAFLD patients without (F0; *n* = 10) and those with (F1-3; *n* = 20) hepatic fibrosis (1.67 ± 0.12 vs. 2.00 ± 0.19; *p* = 0.403), or between NAFLD patients with fibrosis stage F0/F1 (*n* = 24) and F2/F3 (*n* = 6) (1.80 ± 0.14 vs. 2.24 ± 0.39; *p* = 0.178).

**Fig. 2:**
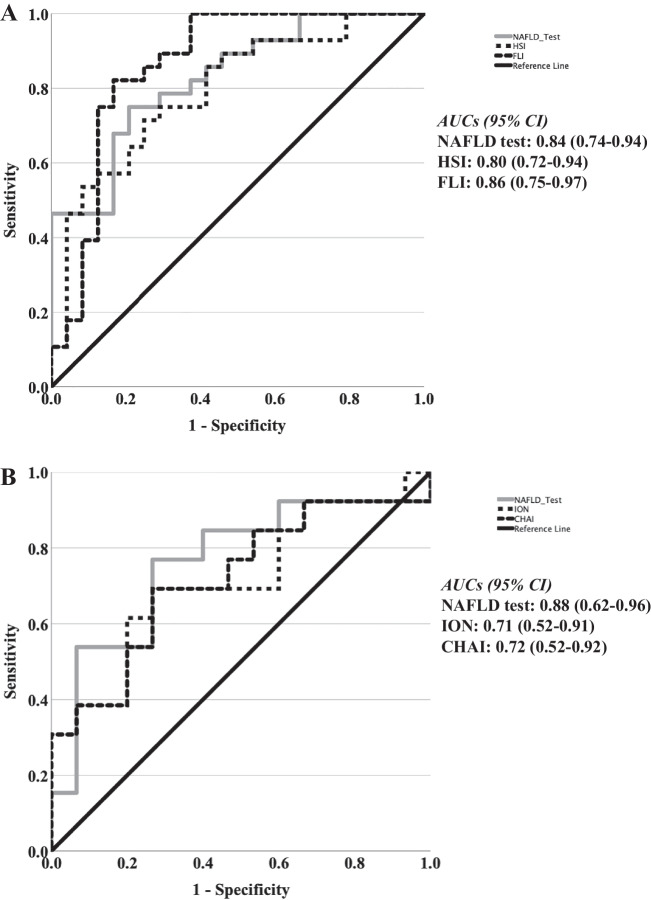
**A** ROC curves for the NAFLD test, FLI, and HSI for the diagnosis of NAFLD vs. non-NAFLD. **B** ROC curves for the NAFLD test, ION, and CHAI for the diagnosis of NASH vs. NAFL. ROC curve analysis was used and the AUC was calculated. The diagonal lines represent the reference lines. Abbreviations: AST, aspartate transaminase; AUC, area under the ROC curve; CHAI, cytokeratin-18/HOMA-IR/AST index; FLI, fatty liver index; CI, confidence interval; HOMA-IR, homeostasis model assessment-insulin resistance; HSI, hepatic steatosis index; ION, index of NASH, NAFLD, nonalcoholic fatty liver disease; NASH, nonalcoholic steatohepatitis; ROC, receiver operating characteristic

In the ROC curve analysis, the AUC of the NAFLD test was 0.84 (95% CI: 0.74–0.94; *p* < 0.001) for differentiation between NAFLD and non-NAFLD controls. For a cut-off of 1.47, the NAFLD test had 77% sensitivity, 79% specificity, 82% positive predictive value (PPV), 73% negative predictive value (NPV), and 78% accuracy for identification of NAFLD. When the performance of the NAFLD test to identify NAFLD was compared with the performance of two other validated non-invasive tests of NAFLD (FLI and HSI), the NAFLD test performed similarly to FLI (AUC: 0.86 [95% CI]: 0.75–0.97; *p* < 0.001) and HSI (AUC: 0.80 [95% CI]: 0.72–0.94; *p* < 0.001; Fig. 2A).

For differentiation between NAFL and NASH patients, the AUC of the NAFLD test was 0.88 (95% CI: 0.62–0.96; *p* = 0.007). For a cut-off of 1.63, the NAFLD test had 80% sensitivity, 73% specificity, 75% PPV, 79% NPV, and 77% accuracy for identification of NASH (vs. NAFL). When the performance of the NAFLD test to identify NASH (vs. NAFL) was compared with the performance of two other previously introduced non-invasive tests of NASH (ION and CHAI), the NAFLD test was shown to perform better than ION (AUC: 0.71 [95% CI]: 0.52–0.91; *p* = 0.056) and CHAI (AUC: 0.72 [95% CI]: 0.52–0.92; *p* = 0.050; Fig. 2B).

The NAFLD test did not provide statistically significant results for the identification of NAFLD patients with F0 vs. F1-F3 (AUC: 0.60 [95% CI]: 0.38–0.81; *p* = 0.386), or of NAFLD patients with F0/F1 vs. F2/F3 (AUC: 0.68 [95% CI]: 0.45–0.91; *p* = 0.125).

## Discussion

In this post-hoc analysis, the NAFLD test was validated as a non-invasive tool for NAFLD in an independent cohort of biopsy-proven NAFLD patients. Of note, this was the first report on the NAFLD test in a European (Greek) population following the introduction of the index in Egyptian, Chinese, and Chilean populations [10]. More specifically, the NAFLD test was able to differentiate between NAFLD patients and non-NAFLD individuals with acceptable accuracy and an AUC (0.84; 95% CI: 0.74–0.94) similar to those reported in its introductory report [10]. The NAFLD test showed similar accuracy to that of other validated indices of NAFLD (vs. non-NAFLD), i.e., FLI and HSI (Fig. 1A).

Importantly, in our study, the NAFLD test was also shown, for the first time, to differentiate between NASH and NAFL patients with acceptable accuracy and higher AUC (0.88; 0.62–0.96) than those of other non-invasive indices previously introduced for the differentiation between NASH and NAFL (CHAI [11] and ION [14]). It should be highlighted that despite the numerous indices introduced for the non-invasive diagnosis of NAFLD (vs. non-NAFLD individuals) or hepatic fibrosis, only limited indices have been introduced for the non-invasive diagnosis of NASH (vs. NAFL) [5]. Thus, this result seems to be of considerable importance, although it requires validation by other independent cohorts.

The NAFLD test could not identify either NAFLD patients without hepatic fibrosis (F0) vs. patients with fibrosis (F1-F3), or NAFLD patients without or early fibrosis (F0/F1) vs. patients with significant/advanced fibrosis (F2/F3). In this regard, we avoided comparing the performance of NAFLD test with other validated indices of hepatic fibrosis, such as the fibrosis-4 index or the NAFLD fibrosis score. To date, no index has provided acceptable accuracy for multiple diagnostic uses, i.e., NAFLD (vs. non-NAFLD), NASH (vs. NAFL), and significant or advanced fibrosis (vs. no or early fibrosis).

The main strengths of this study are the histological confirmation of NAFLD and its validation for the first time in a European population. Some limitations include the relatively small sample size, although it was sufficient to show significant differences between NAFLD and non-NAFLD, as well as between NASH and NAFL. Moreover, the controls did not undergo liver biopsy owing to the above-mentioned ethical considerations. Furthermore, this study was not designed specifically for the validation of the NAFLD test, but it was a *post-hoc* analysis of a previous study [11]. Although the study showed that the NAFLD test performed better in differentiating between NASH and NAFL compared with ION or CHAI, the design of the present diagnostic study does not allow for the drawing of secure conclusions that could explain the superiority of the NAFLD test; this would require studies of a different design (e.g., mechanistic studies). Similarly, the design of this study does not allow for secure conclusions as to the inability of the NAFLD test to differentiate between NAFLD patients with hepatic fibrosis and those without.

In conclusion, the current study has demonstrated that the NAFLD test, an inexpensive and easy-to-calculate index, can distinguish between NAFLD patients and non-NAFLD individuals, as well as between NASH and NAFL patients. The validation of the NAFLD test may provide benefit for the non-invasive diagnosis of this disease, which represents a global health burden; however, this requires further validation by other large-scale independent cohorts, preferably with histological confirmation.

## Data Availability

The raw data of this study are available on reasonable request by the corresponding author.

## References

[1] Polyzos SA, Kang ES, Boutari C, Rhee EJ, Mantzoros CS (2020). Current and emerging pharmacological options for the treatment of nonalcoholic steatohepatitis. Metabolism.

[2] Younossi ZM, Golabi P, de Avila L, Minhui Paik J, Srishord M, Fukui N, Qiu Y, Burns L, Afendy A, Nader F (2019). The Global epidemiology of NAFLD and NASH in patients with type 2 diabetes: a systematic review and meta-analysis. J Hepatol.

[3] Rinella ME, Lazarus JV, Ratziu V, Francque SM, Sanyal AJ, Kanwal F, Romero D, Abdelmalek MF, Anstee QM, Arab JP, Arrese M, Bataller R, Beuers U, Boursier J, Bugianesi E, Byrne C, Castro Narro GE, Chowdhury A, Cortez-Pinto H, Cryer D, Cusi K, El-Kassas M, Klein S, Eskridge W, Fan J, Gawrieh S, Guy CD, Harrison SA, Kim SU, Koot B, Korenjak M, Kowdley K, Lacaille F, Loomba R, Mitchell-Thain R, Morgan TR, Powell E, Roden M, Romero-Gómez M, Silva M, Singh SP, Sookoian SC, Spearman CW, Tiniakos D, Valenti L, Vos MB, Wai-Sun Wong V, Xanthakos S, Yilmaz Y, Younossi Z, Hobbs A, Villota-Rivas M, Newsome PN (2023). A multi-society Delphi consensus statement on new fatty liver disease nomenclature. J Hepatol.

[4] Makri E, Goulas A, Polyzos SA (2021). Epidemiology, pathogenesis, diagnosis and emerging treatment of nonalcoholic fatty liver disease. Arch Med Res.

[5] Kouvari M, Valenzuela-Vallejo L, Guatibonza-Garcia V, Polyzos SA, Deng Y, Kokkorakis M, Agraz M, Mylonakis SC, Katsarou A, Verrastro O, Markakis G, Eslam M, Papatheodoridis G, George J, Mingrone G, Mantzoros CS (2023). Liver biopsy-based validation, confirmation and comparison of the diagnostic performance of established and novel non-invasive steatotic liver disease indexes: Results from a large multi-center study. Metabolism.

[6] Perakakis N, Polyzos SA, Yazdani A, Sala-Vila A, Kountouras J, Anastasilakis AD, Mantzoros CS (2019). Non-invasive diagnosis of non-alcoholic steatohepatitis and fibrosis with the use of omics and supervised learning: A proof of concept study. Metabolism.

[7] Kechagias S, Ekstedt M, Simonsson C, Nasr P (2022). Non-invasive diagnosis and staging of non-alcoholic fatty liver disease. Hormones (Athens).

[8] Polyzos SA, Mantzoros CS (2014). Necessity for timely noninvasive diagnosis of nonalcoholic fatty liver disease. Metabolism.

[9] Athyros VG, Polyzos SA, Kountouras J, Katsiki N, Anagnostis P, Doumas M, Mantzoros CS (2020). Non-alcoholic fatty liver disease treatment in patients with type 2 diabetes mellitus; new kids on the block. Curr Vasc Pharmacol.

[10] Omran M, Omr M, Mohamed AA, Abdelghafour RA, Muharram NM, Hassan MB, Fangry A, Emran T, Arab JP, Arnold J, Diaz LA, Zheng MH, El-Kassas M (2023). Development and validation of nonalcoholic fatty liver disease test: a simple sensitive and specific marker for early diagnosis of nonalcoholic fatty liver disease. Eur J Gastroenterol Hepatol.

[11] Polyzos SA, Kountouras J, Papatheodorou A, Katsiki E, Patsiaoura K, Zafeiriadou E, Papadopoulou E, Zavos C, Terpos E (2013). Adipocytokines and cytokeratin-18 in patients with nonalcoholic fatty liver disease: introduction of CHA index. Ann Hepatol.

[12] Bedogni G, Bellentani S, Miglioli L, Masutti F, Passalacqua M, Castiglione A, Tiribelli C (2006). The Fatty Liver Index: a simple and accurate predictor of hepatic steatosis in the general population. BMC Gastroenterol.

[13] Lee JH, Kim D, Kim HJ, Lee CH, Yang JI, Kim W, Kim YJ, Yoon JH, Cho SH, Sung MW, Lee HS (2010). Hepatic steatosis index: a simple screening tool reflecting nonalcoholic fatty liver disease. Dig Liver Dis.

[14] Otgonsuren M, Estep MJ, Hossain N, Younossi E, Frost S, Henry L, Hunt S, Fang Y, Goodman Z, Younossi ZM (2014). Single non-invasive model to diagnose non-alcoholic fatty liver disease (NAFLD) and non-alcoholic steatohepatitis (NASH). J Gastroenterol Hepatol.

